# Type D lymphomatoid papulosis with pityriasis lichenoides et varioliformis acuta-like features in a child with parvovirus infection: a controversial diagnosis in the spectrum of lymphoid proliferations: case report and literature review

**DOI:** 10.1186/s13052-022-01371-x

**Published:** 2022-10-28

**Authors:** Valeria Calcaterra, Riccardo Cavalli, Giorgio A. Croci, Laura Fiori, Antonella Fabiano, Luisa Lunardon, Maria Antonietta Avanzini, Emilio Berti, Gianvincenzo Zuccotti

**Affiliations:** 1 Department of Internal Medicine, University of Pavia, 27100 Pavia, Italy; 2 Pediatric Department, “V. Buzzi” Children’s Hospital, Via Castelvetro n.52, 20154 Milan, Italy; 3 Pediatric Dermatology Unit, Fondazione IRCCS Ca’ Granda Ospedale Maggiore Policlinico, 20154 Milan, Italy; 4 Pathology Unit, Fondazione IRCCS Ca’ Granda Ospedale Maggiore Policlinico, 20154 Milan, Italy; 5 Cell Factory, Pediatric Hematology Oncology Unit, Fondazione IRCCS Policlinico San Matteo, 27100 Pavia, Italy; 6 Department of Biomedical and Clinical Science, University of Milan, 20157 Milan, Italy

**Keywords:** Lymphomatoid papulosis, Type D, Children, pityriasis lichenoides et varioliformis acuta, Parvovirus B19, Cytokine profile, Lymphoproliferative cutaneous disorders, CD8+/CD30+

## Abstract

**Background:**

Lymphomatoid papulosis (LyP) is a rare condition in pediatrics; LyP histological type D has been reported in only 7 children. The differential diagnosis of LyP in the spectrum of lymphoid proliferation remains controversial.

**Case presentation:**

A 6-year-old boy presented to Emergency Department with a 3-week history of an erythematous papulo-vesicular itchy eruption over the submandibular regions, trunk and extremities. History, symptoms and laboratory tests were unremarkable. SARS-CoV-2 antigen was negative. The clinical suspicion of pityriasis lichenoides et varioliformis acuta (PLEVA) was posed, and topical steroids were introduced. One week after, he returned with an extensive painful scaly papulo-erythematous rash, with some ulcerated and necrotic lesions, and fever; therefore the child was hospitalized. Biochemical results were within reference limits, except for high level of C-reactive protein, aspartate aminotransferase, alanine transaminase and bilirubin. Due to a persistently high fever, systemic corticosteroid treatment was administered, with a good clinical response and an improvement of the skin lesions. Anti-PVB-19 Immunoglobulin M was detected. Elevated levels of IL-6, IL-10 and IFN-γ were also recorded. Five days post-admission, most of the lesions had cleared, and the child was discharged. Methotrexate was started, with a positive response. At skin biopsy a “PLEVA-like” pattern was apparent, with a dense, wedge shaped lymphoid infiltrate featuring epidermotropism and morphologically comprising pleomorphic and blastic cells. The pattern of infiltration was highlighted by immunohistochemical stains, which prove the process to feature a CD8+/CD30 + phenotype, the latter being intense on larger cells, with antigenic loss. Polymerase chain reaction for *T-cell receptor gamma* (*TCRG)* chain clonality assessment documented a monoclonal peak. A diagnosis of LyP type D was favored.

**Conclusion:**

The reported case encompasses most of the critical features of two separated entities—PLEVA and LyP—thus providing further support to the concept of them representing declinations within a sole spectrum of disease. Studying the role of infectious agents as trigger potential in lymphoproliferative cutaneous disorders and detecting novel markers of disease, such as cytokines, could have a crucial impact on pathogenic disease mechanisms and perspective therapies.

## Background

Lymphomatoid papulosis (LyP) is a rare condition within primary cutaneous CD30 + lymphoproliferative disorders [1–3]. LyP is clinically characterized by papules, small nodules with varying degrees of central hemorrhage and necrotic ulceration, and histologically by a dermal infiltrate of atypical CD30 + large T-lymphoid cells [1–4]. Usually, the lesions regress after several weeks or months with topical or systemic therapy, with an overall favorable prognosis [3]. However, LyP is characterized by a chronic course, and it has been rarely associated with progression to secondary lymphoma (mycosis fungoides, anaplastic large cell lymphoma, or Hodgkin’s lymphoma) [5].

According to the 2018 update of the World Health Organization–European Organization for Research and Treatment of Cancer (WHO-EORTC) [5], LyP is classified into different histological subtypes: type A-B-C-D-E; a new subtype characterized by the chromosomal rearrangements involving the DUSP-IRF4 locus on 6p25.3,24; and some even more uncommon variants.

LyP occurs primarily in adults in the third and fourth decades of life and is unusual in children [3]. The most common subtype in pediatric age is type A, and LyP type D has been reported in only 7 pediatric patients younger than 18 years of age [1–4, 6–8]. LyP type D can be a diagnostic challenge for clinicians because it overlaps clinical and histopathological features with other cutaneous tumor and inflammatory cutaneous disorders, such as classic or febrile ulceronecrotic pityriasis lichenoides et varioliformis acuta (PLEVA) [3]. PLEVA is a variant of pityriasis lichenoides (PL). Similarly to LyP, PLEVA is characterized by crops of erythematous macules and papules that can become hemorrhagic, pustular, or necrotic. Patients rarely have systemic signs and PLEVA is usually asymptomatic, although lesions may itch [3]. A classic PLEVA may progress to pityriasis lichenoides with ulceronecrosis and hyperthermia (PLUH), associated with fever and a high mortality risk [3, 8–10].

The hypothesis that LyP and PLEVA could stand within the same spectrum of lymphoid proliferation has been discussed in the literature [8, 9]. Hystologically, a PLEVA variant characterized by a conspicuous CD30^+^ component shows a considerable overlap with LyP [10], leading to a difficult diagnosis. In CD30+-rich variant of PLEVA and in PLUH, an association with PVB-19 has been proposed [10, 11]. However, a common underlying pathogenic mechanism is not clear, and no specific laboratory tests may help in differential diagnosis between Lyp and PLEVA.

We present a case of a child with LyP type D that shares symptoms with PLEVA and in which parvovirus B19 (PVB-19) infection was detected. The clinical course, histopathological and immunophenotyping studies, and cytokine profile are described. A literature review concerning this rare disease in pediatrics and its diagnosis is also discussed.

## Case presentation

### Clinical course

A 6-year-old boy presented to our Emergency Department with a 3-week history of an erythematous papulo-vesicular itchy eruption over the submandibular regions, trunk, and extremities. His overall condition was fine; no fever or constitutional symptoms were recorded. There was no clear precipitating factor, including infections, the use of drugs, vaccines, or food allergies. Past medical and family history were unremarkable.

Laboratory tests, including the complete blood count (white blood cells 5.4 × 10^9^/L with 27.5% neutrophils and 47.9% lymphocytes, hemoglobin 12.2 g/dl, and platelet 323.000/mmc), serum electrolytes, glucose, kidney and liver function, C-reactive protein, coagulation parameters, and standard urinalysis were normal. SARS-CoV-2 antigen detecting test from a nasopharyngeal swab was negative. The clinical suspicion of PLEVA was posed, and topical steroids were introduced. A serologic test to detect the presence of antibodies against viruses (cytomegalovirus, Epstein–Barr virus, PVB-19) were and bacteria (Mycoplasma) and dermatological evaluation were prescribed.

**Fig. 1:**
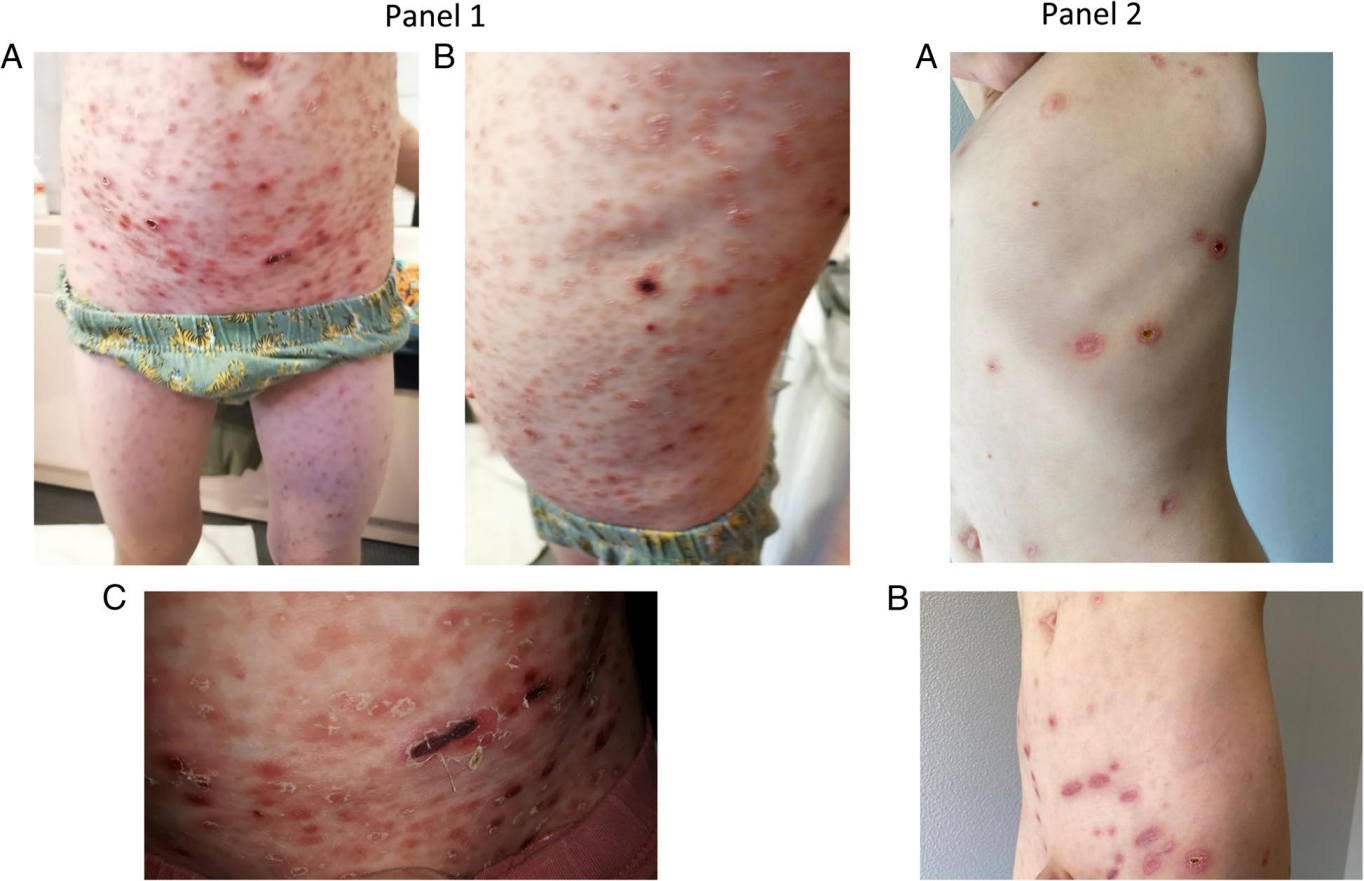
Skin lesions at admission (Panel 1): the red papules with scale, crust, or ulcerations involving the trunk, limbs, flexures at admission (1 **A**, 1**B**); ulcerated and necrotic lesion on the trunk (1 **C**); Clinical response to treatment (Panel 2): the improvement of the lesions on the trunk (2 **A**) and inguinal region (2**B**)

One week after, he returned with an extensive painful scaly papulo-erythematous rash, with some ulcerated and necrotic lesions involving the trunk, limbs, and flexures (Fig. 1, Panel 1). A slight elevation of temperature (37·8 °C) was also noted. No other systemic symptoms, including neurological, pulmonary, cardiac, or gastrointestinal symptoms, were recorded. Antibiotic therapy (amoxicillin–clavulanate 50 mg/kg per day) was prescribed, and topical steroid use was continued.

Due to a worsening fever, the child was then admitted to the Pediatric Department of the “Vittore Buzzi” Children’s Hospital (Milan, Italy). A multidisciplinary care planning with the Pediatric Dermatology Unit, Fondazione IRCCS Ca’ Granda Ospedale Maggiore Policlinico (Milan, Italy), was adopted.

Complete blood count, alkaline phosphatase, lactate dehydrogenase, creatinine, blood urea nitrogen, glucose, creatinine kinase, and urinalysis were persistently within reference limits. A normal level of plasma amino acid was also documented. High level of C-reactive protein (34.8 mg/dl, normal ≤ 10) aspartate aminotransferase (102 U/L, normal 11–34), alanine transaminase (93 U/L, normal ≤ 49) and bilirubin (total 1.35 mg/dl, normal < 1.2; direct 0.53 mg/dl, normal ≤ 0.3) were instead recorded.

A skin biopsy was performed.

Antibiotic therapy was continued. Due to a persistently high fever (> 40 °C), systemic corticosteroid treatment (prednisone 25 mg/kg) was administered, with a good clinical response; the patient became afebrile, and a progressive improvement of the skin lesions was obtained without the appearance of new lesions.

Anti-PVB-19 Immunoglobulin M was detected. Serologies for Mycoplasma, cytomegalovirus, and Epstein–Barr virus were negative.

Five days post-admission, most of the lesions had cleared, and the child was discharged. Methotrexate (7.5 mg/week) was started, with a positive response, Fig. 1, Panel 2. Close long-term monitoring was scheduled.

### Histopathologic examination and immunophenotyping

**Fig. 2:**
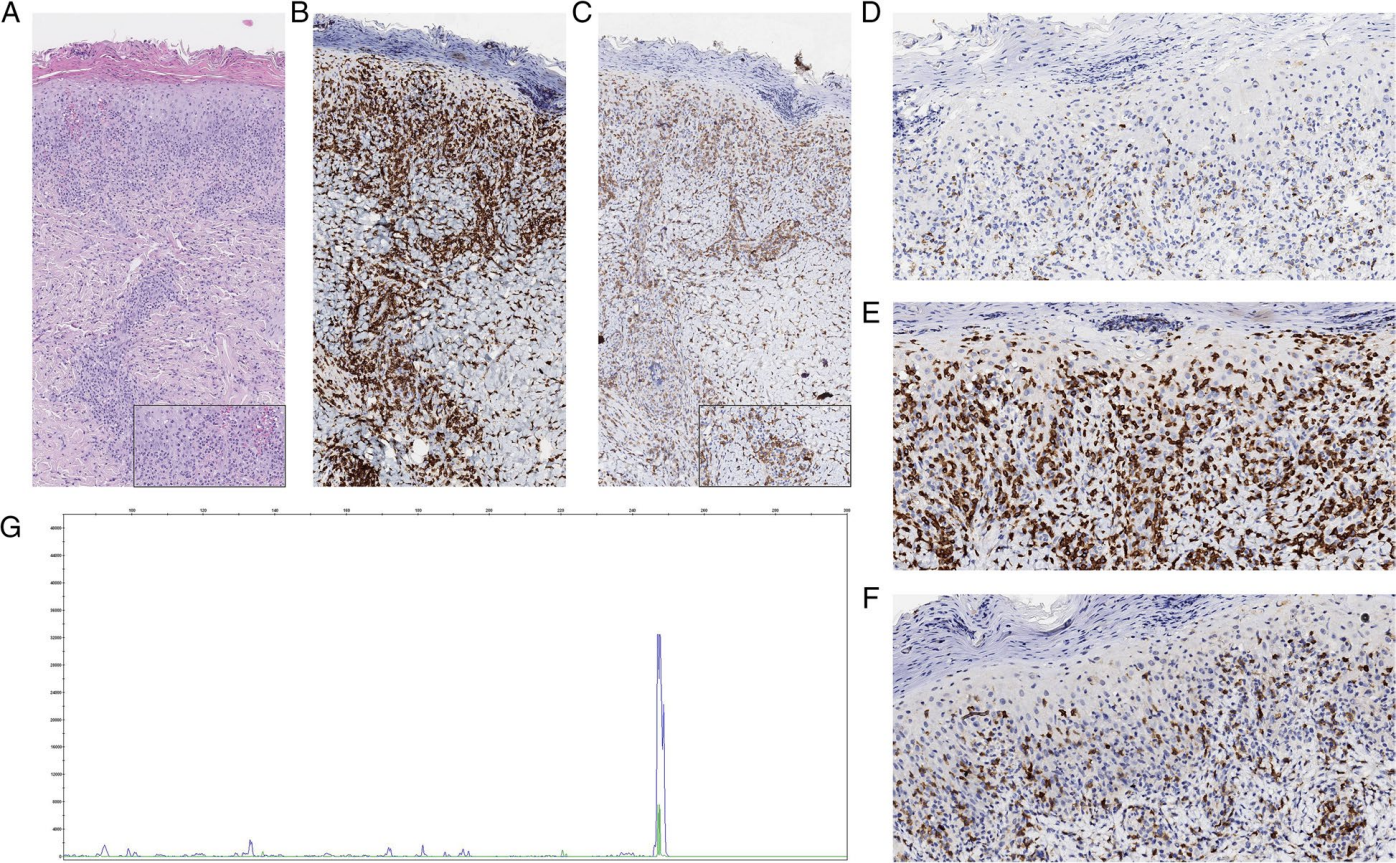
Histology: At lower magnification (Panel a: HE, 100x) a “PLEVA-like” pattern is apparent, with a dense, wedge shaped lymphoid infiltrate featuring epidermotropism and morphologically comprising pleomorphic and blastic cells (Panel a inset: HE 400x). The pattern of infiltration is highlighted by immunohistochemical stains, which prove the process to feature a CD8+/CD30 + phenotype (Panel b: CD8, 100x; Panel c: CD30, 100x), the latter being intense on larger cells (Panel c inset: CD30, 400x), with antigenic loss (Panel d: CD2, 100x; Panel e: CD3, 100x; Panel f: CD5, 100x). GeneScan analysis (Panel g) depicts a monoclonal *TCRG* peak

A skin biopsy performed on admission to the Pediatric Department revealed a “PLEVA-like” morphologic pattern, including features of acanthosis, hyperkeratosis, parakeratosis, and moderate spongiosis of the epidermis, with edema and hemorrhage of the papillary dermis. Adjoining, a few granulocytes and an abundant lymphoid infiltrate were documented, the latter featuring epidermotropism in a pagetoid fashion and extending throughout the dermis in a perivascular and periadnexal configuration, with scant tropism for follicular and eccrine structures. Morphologically, lymphoid cells were medium- to large-sized and slightly pleomorphic, with admixed a minor component of larger blasts. Immunohistochemistry showed an activated, CD8 + cytotoxic, partially defective T-cell phenotype (CD2-/+, CD3+, CD5-, CD7-/+, CD4-, CD8+, perforin+, CD56-, TCR-delta-), with moderate to intense CD30-positivity on 60–70% of the cells (Fig. 2). Polymerase chain reaction for *T-cell receptor gamma (TCRG)* chain clonality assessment, according to BIOMED-2 guidelines, documented a monoclonal peak (Fig. 1, Panel 3G). Besides the clinic-pathologic, PLEVA-like picture, a diagnosis of LyP type D was favored.

### Cytokine profile

Serum levels of tumor necrosis factor (TNF)-α, interleukin (IL)-4-6-10, and interferon (IFN)-γ were measured on admission by using commercially available sandwich enzyme-linked immunosorbent assays (R&D system ELISA kit).

**Fig. 3:**
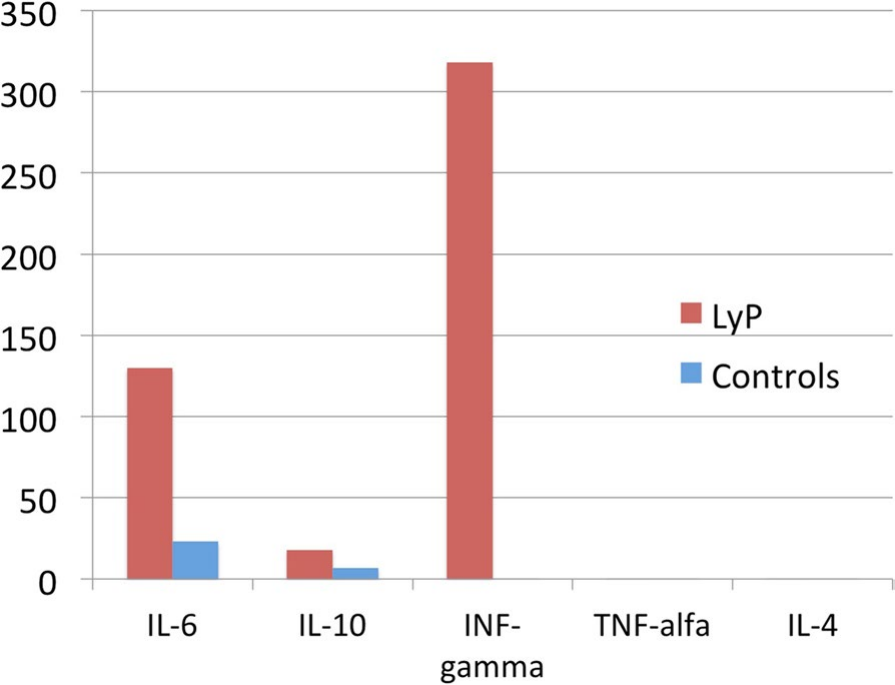
Cytokine profile where elevated levels of IL-6, IL-10 and IFN-γ were showed

Compared to age-matched controls, elevated levels of IL-6 (130 pg/ml; control values 23 ± 16), IL-10 (18 pg/ml; control values 6.6 ± 6.6), and IFN-γ (318 pg/ml; control values < 0.1were recorded. IL-4 and TNF-α were below the detection limit (< 0.1 pg/ml), as in controls (Fig. 3).

## Discussion and conclusions

LyP is a recurrent, self-healing, clinically benign, histologically malignant eruption in the spectrum of CD30 + limphoproliferative cutaneous disorders [4, 9]. According to WHO-EORTC, five main histological subtypes (A-E) has been reported [5]. Immunophenotypically, most LyP cases are derived from CD4-positive T-helper lymphocytes with CD30 antigen co-expression [5]. Type D constitutes a morphological LyP variant, closely mimicking a few conditions with very divergent clinical outcomes, i.e., PLEVA on the indolent side and primary cutaneous CD8-positive aggressive epidermotropic cytotoxic T-cell lymphoma on the very aggressive one [5]. As in our case, the latter condition being ruled out on clinical grounds, LyP versus PLEVA stood open for discussion.

From a clinical perspective, LyP type D is rarely described in children. As reported in Table 1, to date, only seven cases have been previously reported [1–4, 6–8]. Including our patient, the mean age at diagnosis was 10.1 ± 3.1 years, without difference in the sex distribution (4 F/4 M). In most cases, multiple red scaly papules, crust, or necrotic ulceration were described; only two patients showed a single ulcerated nodule.

**Table 1:** Pediatric cases of Lymphomatoid papulosis type D reported in the literature

Case No.	References	Sex	Age	Number of lesions	Type of lesions	Distribution of lesions	Other symptoms	Therapy
1	Brown [1]	F	10	Single	Ulcer	Right upper inner arm	None	Clobetasol propionate Doxycycline
2	Marschalkó et al. [2]	M	12	Multiple	Necrotic, itchy papules	Trunk and extremities	None	UVA
3	Marschalkó et al. [2]	F	5	Multiple	Erythematous necrotic papules	Trunk and lower extremities	None	Topical steroid
4	Barret [3]	M	13	Multiple	Red scaly papules and plaques with focal necrosis and crusting	Trunk, neck, extremities, face, and groin	Severe pain, mild pruritus, fever	Treatment with minocycline and topical steroids
5	Saggini et [4]	F	10	Multiple	Red papules with scale, crust, or ulcerations	Generalized	None	Information not available
6	McQuitty et al. [6]	F	11	Single	Ulcerated nodule	Face	None	Information not available
7	Magro et al. [7]	M	14	Multiple	“Blistering bumps,” ulcernecrotic papules	Head, neck, trunk	Fever, headache, sore throat, myalgia	Bexarotene gel
8	Our case	M	6	Multiple	Red papules with scale, crust, or ulcerations	Trunk, neck, extremities, flexures	Fever, pruritus, pain	Topical and systemic steroid Methotrexate

The clinical presentation of CD8 + LyP is homogeneous and does not seems to be influenced by the diversity of histologic features [3, 5, 7]. Similar to our patient, the cases that Magro et al. [7] and Barret et al. [3] reported also featured a high temperature, supporting that systemic symptoms may be present in LyP type D in patients of pediatric age. However, in our patient, fever might have also been associated with PVB-19 infection.

When integrating the clinical with histopathologic findings, strong arguments could be provided in favor of PLEVA, but the morphology and phenotype fit more within a definition of lymphoproliferative disorder/lymphomatoid reaction. Taking into account published data on such a differential diagnosis, a CD30+-rich variant of PLEVA could be considered, as described by Kempf and coworkers [10], but our case would have fulfilled the exclusion criteria of such a series due to the high number of medium- to large-sized pleomorphic to blastic cells. Furthermore, the clearcut defective T-cell phenotype may overlap the features of the atypical variant of pityriasis lichenoides, from which our case stands apart on the ground of cytologic pleomorphism and, most importantly, CD30 expression [12]. Not helpful for a differential diagnosis, *TCRG* clonality can be consistent either with a WHO-defined T-cell lymphoproliferative disorder or with an antigen-driven, cytotoxic CD8 + T-cell expansion [13, 14]. In our case, the documentation of a clonal peak constitutes a further—albeit weak—indication in support of a LyP-type process, and prospectively, it serves as the benchmark for further monitoring the disease in the contingency of a relapse.

Usually, LyP eruptions evolve and regress within weeks; in some cases, LyP may share a clinical presentation with PL variants, posing a diagnostic challenge. As reported [8, 15], PL, LyP, and cutaneous T-cell lymphoma (CTCL) could be represented as a spectrum of lymphoproliferative disorders; skin biopsy and immunophenotyping remain crucial players in the differential diagnosis of these entities. Our case presented as mimicking febrile ulceronecrotic PLEVA phenotype, with progression of the lesions and hyperthermia; however, the presence of medium- to large-sized atypical CD30 + pleomorphic cells and prominent pagetoid epidermotropism is inconsistent with the PLEVA diagnosis.

An association between PLEVA and PVB-19 has been previously described [15–17], particularly in variants mimicking Lp and other cutaneous lymphomas [10] and in PLUH [11]; in these forms, a perturbation of the immunological homeostasis of keratinocytes with the subsequent activation of TCR-restricted effector cytotoxic T cells has been suggested, and a causative role of PVB-19 should be not excluded [11]. On the contrary, no data on the association between LyP type D and PVB19 have been documented. Considering that PVB-19 has also been proposed as a potential trigger in malignant childhood hemopathy, including lymphoma, in our patient the PVB-19 infection could be very relevant to consider in overlapping and/or transitional forms of lymphoproliferative disorders; further studies will be necessary to consider a causative role of PVB-19 in Lyp.

To date, no specific laboratory tests have been made available for the differential diagnosis of LyP, PL, and CTCL. In the present case, we described a cytokine profile, at admission, characterized by increased levels of IL-6, IL-10, and IFN-γ plasma cytokines; no IL-4 and TNF-α modified expression was found. To the best of our knowledge, this is the first report on the cytokine profile in LyP type D, and the elevated value of IFN-γ seems to be particularly intriguing. An increased IFN-γ has been involved in the explanation for the epidermotropism of CTCL; it induces keratinocytes being able to secrete IFN-γ-inducible protein-l0 (IP-10) and express intercellular adhesion molecules (ICAMs), thus enabling them to attract and retain CD4 + lymphocytes in the epidermis [18]. Antitumor and immunomodulatory functions of IFN-γ have been also well-described in tumor progression and regression [19]. According to Sigurdsson et al., IL-4 and IFN-γ expression were present in the dermal infiltrate of patients with erythroderma and mycosis fungoides, with a difference in the ratio between the two entities [19], illustrating plausible different patho-mechanisms. Additionally, in the literature, increased levels of cytokines, such as TNF-α, IL-2-4-6-10, and IFN-γ in transition from PLEVA to PLUH were also documented [20]. In our case, the effects of PVB-19 on cytokine expression could be also considered. Despite in the present case cytokine profiles at different time points were not available, all these data support the idea that cytokine profiles may be different in different lymphoproliferative disorders, and their role as markers in the differential diagnosis and disease progression cannot be excluded.

Concerning the treatment, most cases of LyP are treated conservatively with topical steroid therapy [1–4, 6–8]; we also used systemic steroids due to a persistently high fever and methotrexate for its antiproliferative, anti-inflammatory, and immunosuppressive properties [21]. The full resolution of symptoms may require a long time. In all reported cases, no malignancy has been reported [1–4, 7].

In conclusion, LyP type D represents a very rare entity in pediatrics. Identifying marked epidermotropism and CD8 and CD30 coexpression on biopsy, in combination with the clinical picture, led to a correct diagnosis. Similar to other subtypes, a good response to treatment is obtained in this variant, and no known progression risk to lymphoma is described; however, a long-term follow-up is necessary. The differential diagnosis of LyP in the spectrum of lymphoid proliferation remains a challenge, and it is necessary to keep the discussion open. The reported case encompasses most of the critical features of two separated entities—PLEVA and LyP—thus providing further support to the (almost, but not yet established) concept of them representing declinations within a sole spectrum of disease. Studying the role of infectious agents as trigger potential in lymphoproliferative cutaneous disorders and detecting novel markers of disease, such as cytokines, could have a crucial impact on pathogenic disease mechanisms and the improvement of their perspective therapies.

## Data Availability

The datasets used and/or analysed during the current study are available from the corresponding author on reasonable request.

## Abbreviations

CTCL: Cutaneous T-cell lymphoma
ICAMs: Intercellular adhesion molecules
IFN: Interferon
IL: Interleukin
IP-10: Inducible protein-10
LyP: Lymphomatoid papulosis
PLEVA: Pityriasis lichenoides et varioliformis acuta
PLUH: Pityriasis lichenoides with ulceronecrosis and hyperthermia
PVB-19: Parvovirus B19
TNF: Tumor necrosis factor
WHO-EORTC: World Health Organization–European Organization for Research and Treatment of Cancer
